# Factor Structures of the Hospital Anxiety and Depression Scale in Cancer: Implications of Comprehensive Confirmatory Analysis on the German Version of the HADS in an Oncological Sample

**DOI:** 10.1002/cam4.71992

**Published:** 2026-06-01

**Authors:** Thilo Dietz, Vera Schiewer, Hildegard Labouvie, Anna Hagemeier, Irena Pjanic, Christoph Herrmann‐Lingen, Michael Kusch

**Affiliations:** ^1^ Chair of Medical Sociology, Faculty of Medicine and University Hospital Cologne Institute of Medical Sociology, Health Services Research, and Rehabilitation Science (IMVR), University of Cologne Cologne Germany; ^2^ Department of Internal Medicine I Faculty of Medicine and Cologne University Hospital, University Hospital Cologne Cologne Germany; ^3^ Institute of Medical Statistics and Computational Biology (IMSB) Medical Faculty and University Hospital Cologne, University of Cologne Cologne Germany; ^4^ Division of Psychosomatic Medicine, Department of Neurology, Inselspital University Hospital Bern Bern Switzerland; ^5^ Department of Psychosomatic Medicine and Psychotherapy University Medical Centre Göttingen Göttingen Germany

**Keywords:** anxiety, cancer, depression, factor analysis, hads

## Abstract

**Background:**

The Hospital Anxiety and Depression Scale (HADS) is a widely used instrument for assessing anxiety and depression in cancer patients. However, the best factor structure for an oncological setting depending on the time of diagnosis is still unclear.

**Aim:**

The objective of this study was to investigate the factor structure of the German‐language version of the HADS in order to review the use of the HADS subscales and the HADS total scale in standard psycho‐oncological care for cancer patients.

**Method:**

A secondary data analysis of *N* = 3395 cancer patients receiving psycho‐oncological care was conducted to analyze 22 of the most common HADS factor models from the literature using confirmatory factor analysis. Confirmatory factor analysis was performed using weighted least squares with mean and variance adjustment (WLSMV). Additionally, measurement invariance (MI) analysis was conducted.

**Results:**

The results indicate that two bifactorial models (Bifactor Model 1: higher‐order factor + Anxiety and Depression subscale; Bifactor Model 2: higher‐order factor + Restlessness, Anxiety, and Depression subscale) exhibit better quality than the original two‐factorial structure (independent anxiety and depression scales) of the HADS (CFI: original structure = 0.970–0.971; Bifactor Model 1 = 0.987–0.988; Bifactor Model 2 = 0.990–0.991; TLI: original structure = 0.964–0.966; Bifactor Model 1 = 0.982; Bifactor Model 2 = 0.985–0.986; RMSEA: original structure = 0.082–0.086; Bifactor Model 1 = 0.065; Bifactor Model 2 = 0.053–0.054). The MI analysis revealed significant differences in performance between configural, metric, and scalar invariance for all three models.

**Conclusion:**

The results support the use of a bifactorial factor structure for the HADS‐D, with the original Anxiety and Depression factors/scales operationalized, as well as an overarching factor in the form of the total sum score.

**Abbreviations**
CFA: confirmatory factor analysis
CFI: comparative fit index
CIO: centrum for integrated oncology
EFA: exploratory factor analysis
HADS: Hospital Anxiety and Depression Scale
HADS‐D: German Version of the Hospital Anxiety and Depression Scale
HADS‐T: Total Sum Score of the Hospital Anxiety and Depression Scale
MI: measurement invariances
ML: maximum likelihood
OLR: orthogonal rotation
OR: oblique rotation
PCA: principal components analysis
QOL: quality of life
RMSEA: root mean square error of approximation
SG: subgroup
TLI: Tucker–Lewis index
VR: varimax rotation
WLSMV: weighted least squares with mean and variances

## 1 Background

In 2022, the global incidence of cancer reached an estimated 20 million cases, resulting in 9.2 million deaths attributable to cancer. A salient point of discussion is the comparatively high cancer prevalence (22.4% of all cancer cases worldwide) among countries with low populations, particularly in Europe (9.6% of the world's population) [1]. The 5‐year prevalence of cancer in Germany is estimated to be approximately 1.9 million cases [2]. The increasing prevalence of cancer is attributed to two major factors: the aging of the population and advancements in medicine. The increase in survival rates has concomitantly raised the probability of developing and living with chronic diseases [3]. This includes the assumption of an increasing prevalence of oncological diseases [1].

Anxiety and depression are among the most common psychological symptoms in cancer patients, regardless of cancer type, disease stage, or treatment phase [4]. The prevalence of anxiety disorders within the first 12 months following cancer diagnosis, irrespective of the specific entity, ranges from 12.2% [5] to 25.3% [6]. The prevalence of mood disorders, specifically major depression (single or recurrent), within the first 12 months following cancer diagnosis, irrespective of the specific entity, ranges from 10.3% [5, 7] to 13.1% [6]. These disorders have a significant impact on patients' quality of life (QoL) [8] and are associated with a poorer prognosis and reduced long‐term cancer survival [9]. Psycho‐oncological interventions can improve the QoL and survival of cancer patients, especially in very prevalent entities [10, 11], by providing targeted support based on reliable and valid assessments [12].

The course of anxiety and depression in individuals after a cancer diagnosis is characterized by heterogeneity. Results indicate persistent distress within the first 12 months after diagnosis [13], while others indicate a variable [14] or more pronounced [15] decline in depression and anxiety [15] over 24 months after diagnosis. The potential influence of cancer treatment can only be linked to a limited extent because there are no specific studies on the average duration of cancer treatment. However, a systematic meta‐analysis indicates that the average duration ranges from 46 days for cervical cancer to 75 days for prostate cancer [16]. Therefore, it can be assumed that these longer treatment periods cause psychological stress in patients.

Diagnosis and treatment of mental health problems follows either a categorical approach using classification systems like ICD‐10/11 or DSM‐5, or a dimensional approach [17] using psychometric tools to assess symptom severity [18]. In psycho‐oncological care, assessment approaches are applied either selectively [19, 20] or in combination [21, 22, 23] to reduce psychological distress in cancer patients through guideline‐based care [24, 25, 26]. Initially, psychometric evaluation is conducted to identify patients in need of support and to assign targeted interventions. This is followed by clinical evaluation using psychiatric classification systems, such as the DSM‐5 or ICD‐10/11 [21, 22, 23, 26]. To ensure accurate recording of distress and the need for help, psychometric evaluation requires well‐tested instruments that are highly objective, reliable, and valid [27].

When developing psychometric survey instruments for guideline‐based evaluation, it is essential to consider valid constructs with only one dimension, for example, “psychosocial distress” [28]. Additionally, multidimensional assessments, such as those related to depressive, anxiety, and other mental disorders [29], should be taken into account.

The construct validity of the instruments is being tested using exploratory factor analysis (EFA) and confirmatory factor analysis (CFA), as well as by correlating with comparable methods of identical or similar constructs [30]. Within the field of psycho‐oncological care, the Hospital Anxiety and Depression Scale (HADS; [31]) is one of the most extensively tested psychometric instruments with regard to its uni‐ and multidimensionality of factor structure.

### 1.1 Psychometric Properties and Factor Structures of the HADS

The HADS is a widely used and well‐validated self‐report instrument for assessing these symptoms in physically ill patients, especially cancer, independent of a formal psychiatric diagnosis [32, 33, 34]. The HADS consists of 14 items divided into two subscales—anxiety and depression—each comprising seven items rated on a 4‐point Likert scale (0–3). Subscale scores range from 0 to 21, with thresholds defined as follows: 0–7 (normal), 8–10 (borderline), and ≥ 11 (abnormal) [35, 36, 37] and alternatively into 0–7 (low) versus 8–21 (high) [33]. Some studies also report a total HADS score (HADS‐T) with its own cut‐offs: 0–14 (normal), 15–21 (borderline), and ≥ 22 (abnormal) [35, 38]. The conversion and standardization of the German‐language HADS (HADS‐D) was carried out by Herrmann‐Lingen et al. [35] using a sample from the general population as well as cardiologically ill patients (see Table 1).

**TABLE 1:** Overview of the models of varying factor structures of the Hospital Anxiety and Depression Scale recorded and investigated in the literature.

Factor structures	No.	Assigned items	Samples	Theoretical model	Method	References
1. Factorial model	I	All items: 1, 2, 3, 4, 5, 6, 7, 8, 9, 10, 11, 12, 13, and 14	Cancer patients (*N* = 210)	Negative affectivity [39]; psychological/emotional distress [40]	EFA w/ OR	Razavi et al. [40]
2. Factorial models	II	Anx.: 1, 3, 5, 7, 9, 11, and 13 Dep.: 2, 4, 6, 8, 10, 12, and 14	Cancer patients (*N* = 901) Cardiologic patients (*N* = 1.887)	Emotional distress in form of anxiety and depression [31, 32]	CFA w/ VR	Hinz et al., Zigmond and Snaith, Herrmann‐Lingen et al. [31, 35, 41]
	III.i	Anx.: 1,3, 5, 9, 11, and 13 Dep.: 2, 4, 6, 8, 10, 12, and 14 [*Exclusion of Item 7*]	Cancer patients (*N* = 3.260)		CFA w/ ML	Zeilinger et al. [34]
	III.ii	Anx.: 1,3, 5, 9, 11, and 13 Dep.: 2, 4, 6, 8, 12, and 1 [*Exclusion of Items 7 and 10*]				
	IV	Anx.: 1, 3, 5, 7, 9, 11, and 13 Dep.: 2, 4, 6, 7, 8, 10, 12, and 14 [*Double factor loading for Item 7*]	Cancer patients (*N* = 568)		EFA (PCA) w/ OR	Moorey et al. [42]
	V	Anx.: 1, 3, 5, 7, 9, and 13 Dep.: 2, 4, 6, 10, 12, and 14 [*Exclusion of Items 8 and 11*]	Patients with motor neuron disease (*N* = 298)	NA	Rasch‐Analysis	Gibbons et al. [43]
	VI	Anx.: 1, 3, 5, 9, 11, and 13 Dep.: 2, 4, 6, 10, 12, and 14 [*Exclusion of Item 7 and 8*]	Dementia patients (*N* = 268)		CFA w/ ML	Stott et al. [44]
	VII.i	*Myocardial infarction sample* (*for survey after 6 months*)*:* Anx.: 1, 3, 4, 5, 7, 9, 11 und 13 Dep.: 2, 6, 8, 10, 12 und 14	Patients with myocardial infarction (*N* = 108) Apoplexy patients (*N* = 68)	Anxiety and depression [31]	EFA (PCA) w/ VR; CFA	Johnston [45]
	VII.ii	*Myocardial infarction sample* (*for survey after 12 months*)*:* Anx.: 1, 3, 5, 9, 11, and 13 Dep.: 2, 4, 6, 7, 8, 10, 12, and 14				
	VII.iii	*Apoplexy sample* (*within the first month of illness*)*:* Anx.: 1, 3, 9, 11, and 13 Dep.: 2, 4, 5, 6, 7, 8, 10, 12, and 14				
	VII.iv	*Apoplexy sample* (*within the 6 month of illness*)*:* Anx.: 1,3,5, 6, 9,11, 12, and 13 Dep.: 2, 4, 7, 8, 10, and 14				
3. Factorial models	VIII	Autonomous anx.: 3, 9, and 13 Anhedonic dep.: 2, 4, 6, 8, 10, 12, and 14 Negative affectivity: 1, 5, 7, and 11	General population (*N* = 2.547)	Tripartite theory (negative affectivity, autonomic anxiety, anhedonia) of anxiety and depression [46]	CFA w/ ML	Dunbar et al. [47]
	IX	Anx.: 1, 3, 5, 9, and 13 Dep.: 2, 4, 6, 8, 10, and 12 Restlessness: 7, 11, and 14	Healthy students (*N* = 195)		CFA w/ WLSMV	Caci et al. [48]
	X	Anx.: 3, 5, 9, and 13 Dep.: 2, 4, 6, 8, 10, 12, and 14 Psychomotoric agiation: 1, 7, and 11	Patients with major depression (*N* = 2.669)		EFA (PCA) w/ OR	Friedman et al. [49]
	XI	Anx.: 3, 5, 9, and 13 Psycho‐motoric: 1, 6, 7, 8, 11, and 14 Dep.: 2, 4, 10, and 12	Patients with traumatic brain injury (*N* = 371)		EFA w/ OR; CFA w/ ML	Skilbeck et al. [50]
	XII	Autonomous anx.: 3, 9, and 13 Negative affectivity: 1, 7, and 11 Anhedonic dep.: 2, 4, 6, 8, 10, 12, and 14 [*Exclusion of Item 5*]	Dementia patients (*N* = 284)		CFA w/ ML	Stott et al. [51]
	XIII	Anx.: 3, 5, 9, and 13 Dep.: 2, 4, 6, 8, 10, and 12 Restlessness: 1, 7, 11, and 14	Cancer patients (*N* = 273)	NA	EFA (PCA) w/ OR	Brandberg et al. [52]
	XIV	Factor 1: 3, 5, 8, 9, and 13 Factor 2: 1, 7, 11, 12, and 14 Factor 3: 2, 4, and 6 [*Exclusion of Item 10*]	Dementia patients (*N* = 117)	NA	EFA (PCA) w/ OR; CFA w/ ML	Lewis [53]
4. Factorial models	XV	Well‐being: 4, 10, and 12 Current anxiety: 3, 5, 7, 8, 9, and 13 Power to relax: 1, 6, and 14 Nonspecified factor: 2 and 11	General population (*N* = 163)	NA	EFA (PCA) w/ OR	Andersson [54]
	XVI	Anx.: 1, 5, 7, 11, and 13 Dep.: 2, 4, 6, 12, and 14 Factor 3: 3 and 9 Factor 4: 2 and 8	Patients with advanced cancer and metastasis (*N* = 100)	NA	EFA (PCA) w/ OLR	Lloyd‐Williams et al. [55]
Bifactorial models	XVII.i	Overarching factor: all items Anx.: 1, 3, 5, 7, 9, 11, and 13 Dep.: 2, 4, 6, 8, 10, 12, and 14	Meta‐analysis (*N* = 21.820)	General distress with autonomic anxiety and anhedonic depression [39, 46]	Meta‐CFA	Norton et al. [56]
	XVII.ii	Overarching factor: all items Anx.: 1, 3, 5, 9, and 13 Dep.: 2, 4, 6, 8, 10, and 12 Restlessness: 7, 11, and 14				

Abbreviations: Anx. = anxiety, CFA = confirmatory factor analysis, Dep. = depression, EFA = exploratory factor analysis, ML = maximum likelihood, OLR = orthogonal rotation, OR = oblique rotation, PCA = principal components analysis, VR = varimax rotation, w/ = with, WLSMV = weighted least squares with mean and variances.

Further, the instrument is widely used in psycho‐oncological research and practice and has been subjected to extensive psychometric testing on cancer patients both national [57, 58, 59] and international [38, 60, 61, 62]. In psycho‐oncological practice, the HADS is recommended for the early detection of anxiety and depression as well as psychological distress despite an existing recommendation to test the practical benefits of the HADS [22, 26, 58, 59, 60]. In addition, there is a partial rejection of its use, as the various validation studies of the survey instrument and its identified factor structure depend on the samples surveyed and the statistical methods used to validate the factor structure [63].

Since its initial publication, the factor structure of the HADS has been examined in a wide range of studies. The published results ([34, 40, 41, 42, 43, 44, 45, 47, 52, 54, 55, 64, 65, 66]; cf. Table 1) have included various factor structures, ranging from one to four factors and also including bifactorial models. A comprehensive review by Cosco et al. [67] analyzed 50 studies, where the majority of the studies (50%) supported a two‐factor solution, followed by an identified three‐factor solution (34%). Unlike Coyne and van Sonderen [63], who contend that the HADS exhibits inconsistent performance across samples, Cosco et al. [67] propose that more recent analyses of the HADS's factorial quality are warranted. Norton et al. [56] conducted a meta‐CFA, which revealed that a bifactor model using the original HADS subscales and a superordinate factor from all items demonstrated the best model fit. This result has been replicated in subsequent studies [68, 69]. The most recent analysis of the HADS factor structure by Lloyd et al. [66] confirms its original structure using both a Rasch analysis and an EFA using PCA and subsequent CFA. Several recent factor analytic studies on the HADS confirm the bifactorial structure findings [70, 71].

Recent studies have indicated the HADS's suitability for a transdiagnostic approach [70]. The transdiagnostic approach endeavors to supplant conventional diagnostic systems (ICD or DSM), with the objective of transcending diagnostic boundaries to comprehensively capture fundamental biopsychosocial processes [72]. Transdiagnostic interventions are to be applied across different mental disorders [73] in order to improve clinical effectiveness [74, 75]. To date, there are no studies that have examined the application of the HADS in a transdiagnostic approach with regard to cancer patients.

### 1.2 Aim of the Present Work

Given the widespread use of the HADS‐D as a screening tool for anxiety and depression in cancer patients [23, 26], this study aims to evaluate previously proposed factor structures by applying them to the German version of the instrument. The primary objective is to determine which model best fits oncology populations. This study also addresses a gap in the literature, as a comprehensive analysis of the German HADS has not yet been conducted.

## 2 Methods

### 2.1 Samples and Total Date Set

This study is based on data from psycho‐oncological standard care in clinical psycho‐oncology at the Center for Integrated Oncology (CIO) at the University Hospital of Cologne from February 2009 to April 2021 [23, 76, 77]. Data collection and analysis were conducted based on positive ethics committee votes (AZ 15‐048; AZ 18‐092) for the individual primary studies (due to the privacy policies governing the initial studies and data collection, it is not possible to disclose the data. Please refer to the “Data Availablity Statement” for more information).

**FIGURE 1:**
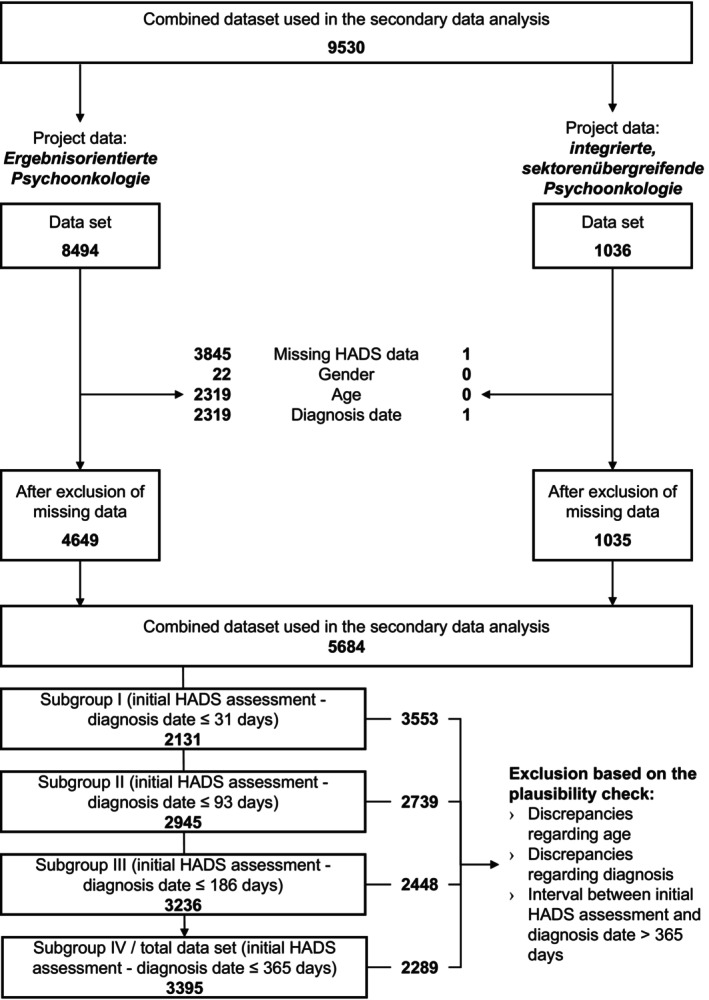
Data consolidation and exclusion of missing data (project translation: *Ergebnisorientierte Psychoonkologie* = outcome‐oriented psycho‐oncology; *integrierte, sektorenübergreifende Psychoonkologie* = integrated psycho‐oncology across care sectors).

The data from both projects were merged and harmonized for targeted analysis (Figure 1). Cases with missing values in any of the following variables were removed from the dataset: (1) complete information in all HADS items or (2) sociodemographic variables (age and gender). An additional exclusion criterion was the HADS assessment > 365 days after diagnosis date. The final adjusted dataset comprised *N* = 3395 cases.

### 2.2 Formation of Subgroups

Given the heterogeneity of the results in the extant literature (cf. [13, 14, 15]), a subgroup analysis was employed to examine the consistency of the factor structure of the HADS during the initial 12 months following diagnosis. The variability of anxiety and depression during this period was addressed by analyzing and identifying the model that best fit the data, depending on the time of data collection after diagnosis. Stratified analyses were conducted in subsamples of the total data set (*N* = 3395) (see Figure 1), which underwent further categorization as outlined below:

*Subgroup I* (*SG* ≤ *1 month*)*:* Initial HADS assessment within the first month after cancer diagnosis (≤ 31 days).

*Subgroup II* (*SG* ≤ *3 months*)*:* Initial HADS assessment within the first 3 months after cancer diagnosis (≤ 93 days).

*Subgroup III* (*SG* ≤ *6 months*)*:* Initial HADS assessment within the first 6 months after cancer diagnosis (≤ 182 days).

*Subgroup IV* (*SG ≤ 12 months*)*:* Initial HADS assessment within the first year after cancer diagnosis (≤ 365 days).

### 2.3 Statistical Methods

The analysis of the individual models identified in Tables 1 and S1 was carried out in a step‐by‐step procedure.

1. First, a descriptive analysis of the data was performed, including testing for normal distribution using the Shapiro–Wilk test.
2. Then, the CFA was calculated for all models within the individual subgroups.
3. Finally, measurement invariance (MI) was calculated for the selected models.

Descriptive and inferential statistics were calculated using IBM SPSS version 30 [78]. The individual models and MI calculations for CFA were performed using R (version 4.4.1) [79] with the Lavaan [80] and semTools [81] packages.

#### 2.3.1 Structural Equation Modeling

The HADS is based on hierarchical models of anxiety and depression [82], which form the basis of CFA and item assignments (see Table 1). According to the integrative hierarchical model of psychopathology (IHM; [82]), a general, higher‐order factor of “general distress” and “negative affectivity” underlies both depressive and anxiety disorders. The tripartite model [46] provides a theoretical reference model for anxiety and depression, which is supported by Cosco et al. [67] and Dunbar et al. [47]. Detailed information on the tripartite model can be found in the Supporting Information Box 1.

The selection of the appropriate estimator in CFA is a topic extensively covered in the literature [34, 83]. Given the nonnormal distribution of the items and the ordinal 4‐point Likert scale of the HADS response options, the weighted least squares method of estimation with means and variances (WLSMV) was selected in accordance with the recommendations of the literature [83, 84, 85].

Model quality was assessed using the comparative fit index (CFI), the Tucker–Lewis index (TLI), and the root mean square error of approximation (RMSEA). MI was examined using the total data set (*N* = 3395; SG IV). MI was evaluated using *χ* ^2^ tests and changes in the CFI. A lack of MI was indicated by a ΔCFI > −0.01 and a ΔRMSEA > 0.015 (cf. [86]).

In accordance with the study objectives, all factor structures of the HADS were analyzed. A stepwise selection process was employed to select the best‐fitting model. Initially, each model was evaluated for adherence to the model fit indices (CFI/TLI > 0.9 [87]; RMSEA < 0.1 [88]). However, if a CFI value of 0.9 or higher was achieved for all models, selection based solely on the CFI would be methodologically inadmissible (cf. [88, 89]). In such cases, the consistency of the RMSEA was considered as an additional basis for decision‐making, as it reflects the absolute discrepancy of the SEM. Consequently, the range of the RMSEA was calculated across the subgroup analyses of the models using the following formula:

$${\text{Range}}_{\text{RMSEA}}=\max\left(\text{RMSEA}\right)-\min\left(\text{RMSEA}\right)$$

Minor discrepancies between subgroups were considered indicative of a high degree of consistency and were operationalized as an additional indicator. In cases where results were identical or similar, the final selection of the model with the best fit was made when a “close fit” was achieved, as defined by a RMSEA value below 0.05 [88, 90].

## 3 Results

### 3.1 Descriptive Statistics and Item Parameters

The majority of patients in all four subgroups were female (60.5% – 58.1%; Table S2). The mean age (±SD) ranged from *M* = 55.51 (±14.31) years (SG I) to *M* = 55.73 (±14.45) years (SG IV). The most common diagnostic groups within the subgroups and the total data set were malignant neoplasms of the breast (C50) with 18.1% (*N* = 613; total data set) to 20.6% (*N* = 445; SG I), followed by malignant neoplasms of the lymphatic, hematopoietic, and related tissues (C81–C96) with 17.8% (*N* = 603; total data set) to 20.1% (*N* = 427; SG I), and in the third place malignant neoplasms of the digestive organs (C15–C26) with 11.3% (*N* = 240; SG I) to 14.9% (*N* = 507; total data set) (see Table S2). Comprehensive details concerning patient characteristics are obtained in Table S1.

Item analysis and Shapiro–Wilk test revealed that all HADS items failed to meet the assumption of normality across the subgroups (see Table S3). Mean values ranged from *M* = 0.53 (I10) to *M* = 1.65 (I3), predominantly skewed to the right with moderate skews (> |0.5|). Comprehensive details concerning item mean, skewness, and normal distribution are obtained in Table S3.

Considering the total sample (SG IV; *N* = 3395), the mean of the anxiety scale was *M* = 8.77 (SD = 4.6; range: 0–21), and the mean value of the depression scale was *M* = 7.02 (SD = 4.76; range: 0–21). The HADS‐T mean was *M* = 15.79 (SD = 8.65; range: 0–42). In total, 59% (*N* = 2004) of cancer patients achieved values > 7 on the anxiety scale and 41% (*N* = 1393) on the depression scale; 1779 patients (54.4%) achieved values > 14 on the HADS‐T. Significant gender differences were identified for the anxiety scale (*T* = −8.315; df = 3393; two‐tailed *p* < 0.001; 95% CI.: −1.628 – −1.007; *male patients*: *M* = 8.01; SD = 4.53; *N* = 1421; *female patients*: *M* = 9.32; SD = 4.57; *N* = 1974) and the HADS‐T (*T* = −4.869; df = 3393; two‐tailed *p* < 0.001; 95% CI.: −2.049 – −0.873; *male patients*: *M* = 14.94; SD = 8.79; *N* = 1421; *female patients*: *M* = 16.4; SD = 8.51; *N* = 1974) between female and male cancer patients. Furthermore, a significant difference in the mean value of the depression scale (*T* = −5.021; df = 1214; *p* < 0.001; 95% CI.: −1.82 to –0.8; C50: *M* = 5.02; SD = 4.46; *N* = 613; C81–C96: *M* = 7.33; SD = 4.64; *N* = 603) and the HADS‐T (*T* = −2.378; df = 1214; *p* = 0.018; 95% CI.: −2.04 to –0.2; C50: *M* = 14.89; SD = 8.28; *N* = 613; C81–C96: M = 16.01; SD = 8.13; *N* = 603) was identified between the two most common diagnosis groups (C50 vs. C81–C96).

### 3.2 Confirmatory Factor Analysis and Selecting ‘Best’ Model

A total of 22 models from 17 key publications (Table 1) were analyzed. Overall, all models achieved a CFI and TLI greater than 0.9 (see Tables 2 and S1). Across all subgroups, Models I and XV had an RMSEA ≥ 0.1 (Tables 2 and S1), thus failing to meet the requirements for model quality [88]. Almost all models, except model XVII.ii (SG I: *p* = 0.176; SG II: *p* = 0.065) achieved significant RMSEA values (*p* < 0.05). Detailed information on each model within each subgroup can be found in Table S1.

**TABLE 2:** Overview of model fit indices and RMSEA‐range across all models.

Model	Range of values			${\text{Range}}_{\text{RMSEA}}$	Fulfillment of all model fit criteria?	RMSEA—close fit (≤ 0.05)?
	CFI	TLI	RMSEA			
I	0.937–0.940	0.926–0.930	0.120–0.121	0.001	No	No
II	0.970–0.971	0.964–0.966	0.082–0.086	0.004	Yes	No
III.i	0.982–0.984	0.978–0.979	0.065–0.068	0.003	Yes	No
III.ii	0.983–0.984	0.979–0.980	0.068–0.071	0.003	Yes	No
IV	0.979–0.980	0.975–0.976	0.061–0.071	0.01	Yes	No
V	0.975–0.977	0.968–0.972	0.084–0.090	0.006	Yes	No
VI	0.986–0.987	0.982–0.983	0.061–0.065	0.004	Yes	No
VII.i	0.945–0.946	0.934–0.936	0.114–0.115	0.001	No	No
VII.ii	0.974–0.975	0.969–0.970	0.078–0.079	0.001	Yes	No
VII.iii	0.949–0.952	0.939–0.943	0.109–0.109	0	Yes	No
VII.iv	0.945–0.974	0.935–0.939	0.112–0.113	0.001	No	No
VIII	0.975–0.975	0.969–0.970	0.077–0.079	0.002	Yes	No
IX	0.977–0.978	0.972–0.973	0.072–0.076	0.004	Yes	No
X	0.979–0.980	0.974–0.975	0.071–0.072	0.001	Yes	No
XI	0.972–0.974	0.965–0.968	0.081–0.083	0.002	Yes	No
XII	0.978–0.980	0.973–0.975	0.073–0.073	0	Yes	No
XIII	0.978–0.979	0.974–0.974	0.072–0.073	0.001	Yes	No
XIV	0.963–0.967	0.953–0.958	0.098–0.101	0.003	No	No
XV	0.958–0.958	0.946–0.957	0.102–0.105	0.003	No	No
XVI	0.972–0.974	0.962–0.965	0.088–0.092	0.004	Yes	No
XVII.i	0.987–0.988	0.982–0.982	0.060–0.060	0	Yes	No
XVII.ii	0.990–0.991	0.985–0.986	0.053–0.054	0.001	Yes	Yes

*Note:* Model assignment according to Table 1; the model fit indices summarized in the table (TLI, CFI, and RMSEA) encompass analyses of all four subgroups—detailed information on individual models for each dataset (subgroup) can be found in Table S1.

Abbreviations: CFI = comparative fit index; TLI = Tucker–Lewis index; RMSEA = root mean square error of approximation.

According to the selection process and the information presented in Tables 2 and S1, Model XVII.ii was selected as the HADS factor model with the best model properties across all subgroup analyses, characterized by a consistent RMSEA with values ranging from 0.053 to 0.054 (${\text{Range}}_{\text{RMSEA}}$ = 0.001). This model was then compared with the original factor structure (Model II) and the bifactorial model with a superordinate factor (Model XVII.i) in terms of MI, allowing for an assessment of the equivalence of the factor loadings and intercepts across the different models. Detailed information on each model within each subgroup can be found in Table S1.

### 3.3 Total Variance and Measurement Invariance of Models

An analysis of the three models (II, XVII.i, and XVII.ii) revealed an overall variance of 0.568–0.622 across all subgroups. The total variance increased with increasing number of patients. Model XVII.ii achieved the highest total variance within all subgroups, ranging from 0.573 to 0.622. Detailed information on the model variances of all three models within each subgroup is reported in Table S4.

Table S5 contains detailed information on MI for all three models. The MI analysis revealed significant differences in performance between configural, metric, and scaled invariance for all three models (Table S5). Examination of MI across male and female patients revealed acceptable goodness‐of‐fit values for configurational and metric invariance (*Model II*: ΔCFI = 0.013; ΔRMSEA = −0.024; *Model XVII.i*: ΔCFI = 0.006; ΔRMSEA = −0.023; *Model XVII.ii*: ΔCFI = 0.005; ΔRMSEA = −0.019) as well as the metric and scaled invariance (*Model II*: ΔCFI = −0.008; ΔRMSEA = 0.007; *Model XVII.i*: ΔCFI = −0.003; ΔRMSEA = 0.002; *Model XVII.ii*: ΔCFI = −0.003; ΔRMSEA = 0.006; Table S5). Detailed information on MI for all three models can be found in Table S5.

In addition, configural‐metric (*Model II*: ΔCFI = 0.004; ΔRMSEA = −0.007; *Model XVII.i*: ΔCFI = −0.004; ΔRMSEA = 0.004; *Model XVII.ii*: ΔCFI = −0.006; ΔRMSEA = 0.01) and metric‐scaled (*Model II*: ΔCFI = −0.007; ΔRMSEA = 0.003; *Model XVII.i*: ΔCFI = −0.003; ΔRMSEA = 0.002; *Model XVII.ii*: ΔCFI = 0; ΔRMSEA = −0.003) invariance were identified for all three models in relation to the differentiation between the two largest diagnostic groups (see Table S5).

Grouping patients into low vs. moderate and moderate vs. high distress resulted in the failure to meet specifications for all three models, except for configural and metric invariance for model XVII.i (ΔCFI = 0.005, ΔRMSEA = 0.009; see Table S5).

### 3.4 Reliability, Factor Loadings, and Covariance

ESM Table E6 provides an overview of the reliability depending on the examined model and subgroup. The results indicated good to very high internal consistency for the scales of the original HADS‐D factor structure (*anxiety*: *α* = 0.847–0.85; *depression*: *α* = 0.866–0.87) and for the overarching factor of models XVII.i and XVII.ii (α = 0.908–0.911). However, the reliability of the factor/scale “Restlessness” of model XVII.ii was unacceptable, with values ranging from *α* = 0.589 to 0.605 (Table S6).

**FIGURE 2:**
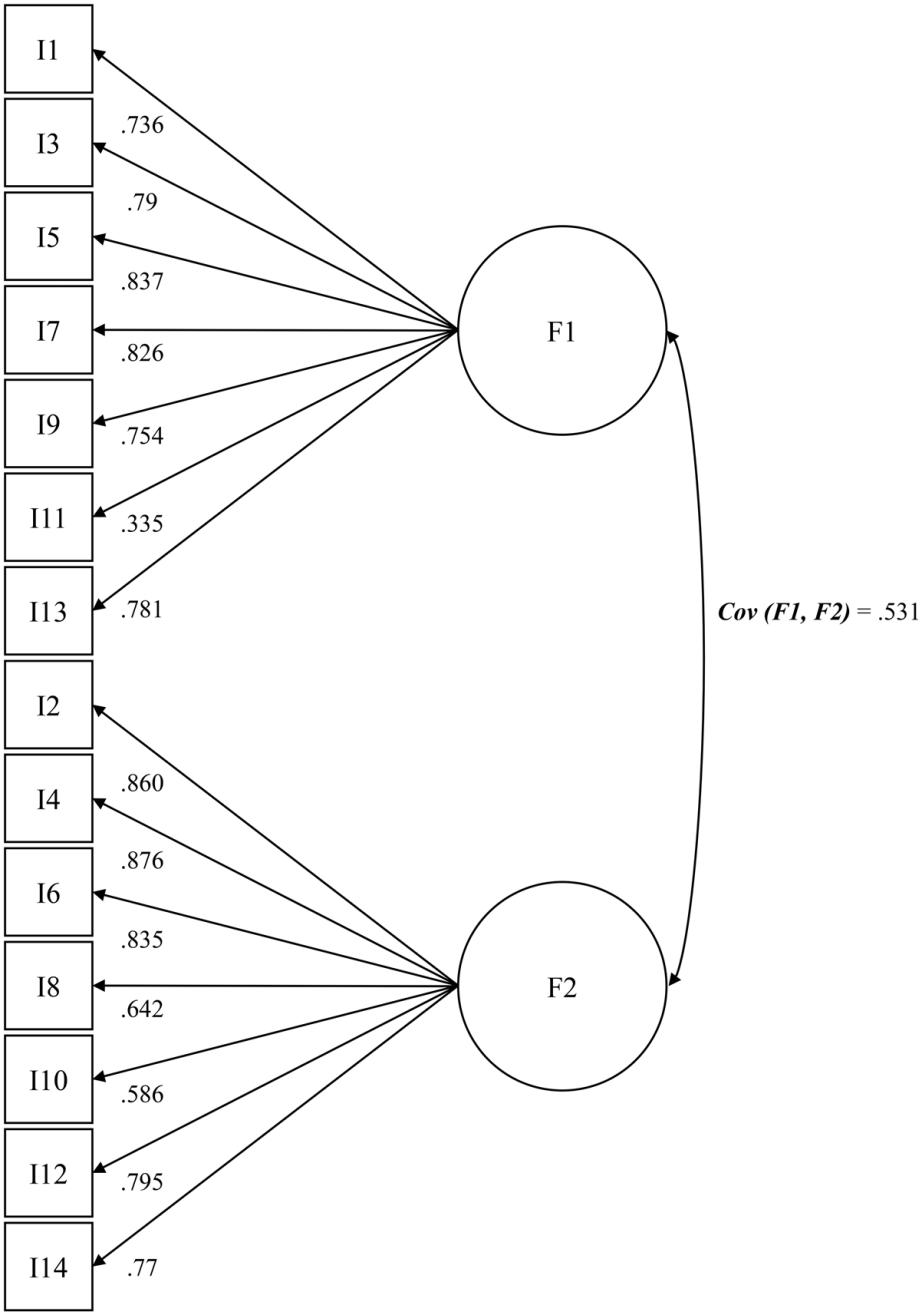
Factor model II [31, 35, 41] incl. standardized regression weights and covariance (SG IV *N* = 3395). Anx. = anxiety; Dep. = depression.

**FIGURE 3:**
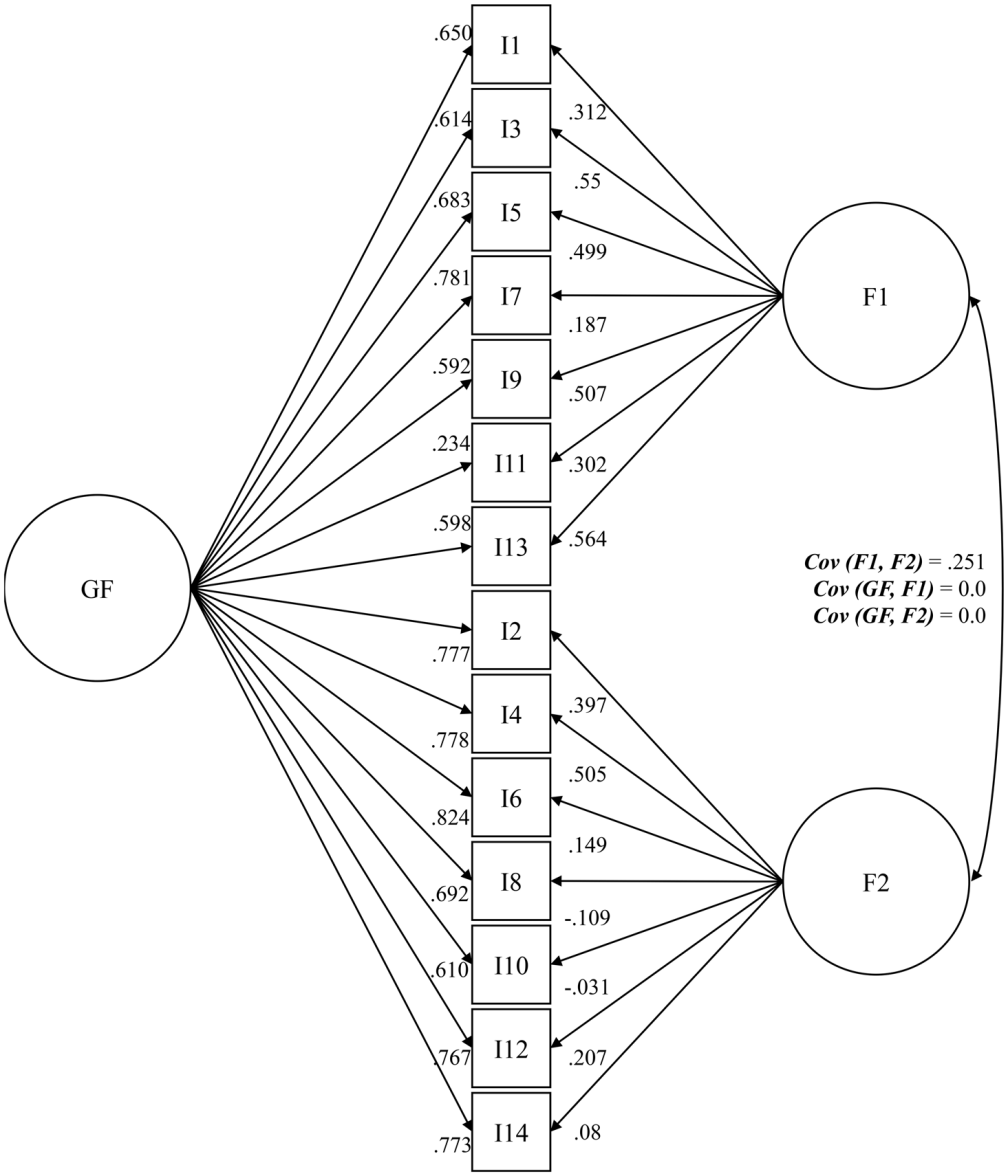
Factor model XVII.i [56] incl. standardized regression weights and covariance (SG IV *N* = 3395). Anx. = anxiety; Dep. = depression; OF = overarching factor.

**FIGURE 4:**
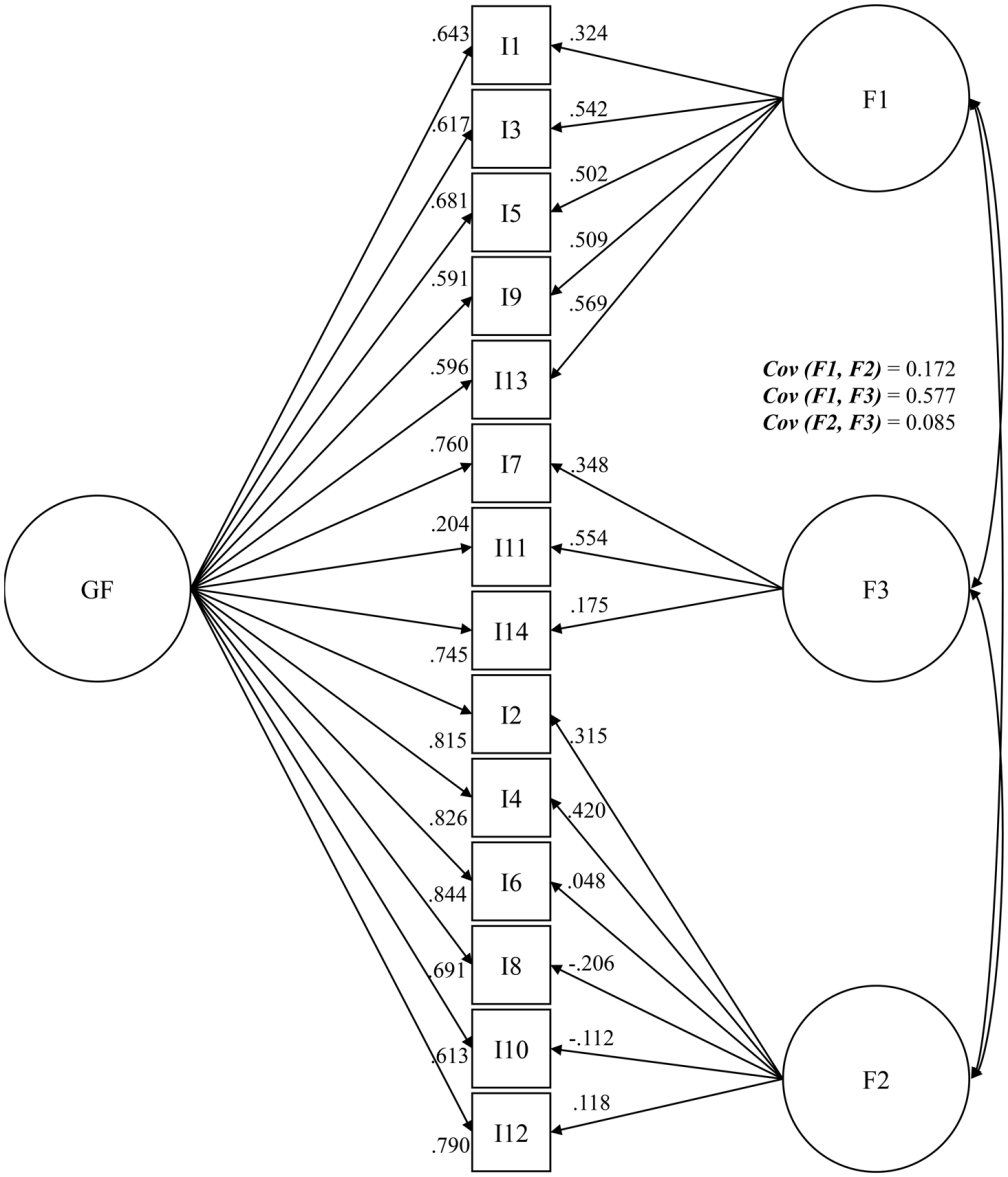
Factor model XVII.ii [56] incl. standardized regression weights and covariance (SG IV *N* = 3395); Anx. = anxiety; Dep. = depression; OF = overarching factor; Restless = restlessness.

Figures 2, 3, 4 illustrate the factor loadings of the three models examined in detail. Model II showed positive covariances between both factors. The loadings of the anxiety factor were mostly very good (*λ* = 0.586 to 0.876), except for item 11, which had a comparatively weak loading of *λ* = 0.335. The loadings of the factor Depression achieved values ranging from acceptable to very good for all items. The detailed factor structure, including factor loadings and covariance, is shown in Figure 2.

Model XVII.i showed a lower covariance between the factors anxiety and depression than Model II. The factor loadings of the overarching factors were mostly high (*λ* = 0.598–0.824), except for Item 11. However, compared to Model II, comparatively lower loadings were identified for the original factors anxiety and depression. Loadings for the factor depression achieved mostly weak (*λ* = 0.08–0.397) and partly negative (I8 = −0.109; I10 = −0.031) loadings, except for Item 4 (*λ* = 0.505). The detailed factor structure, including factor loadings and covariance, is shown in Figure 3.

Model XVII.ii showed similar results to Model XVII.i, with acceptable to very good factor loadings for the overarching factor. However, only moderate covariance was identified between the factors anxiety and restlessness. Furthermore, weak (I2 = 0.315, I6 = 0.048, and II12 = 0.118) to negative (I8 = 0.206; I10 = 0.112) factor loadings were identified for items of the factor depression. The factor restlessness also achieved weak loadings for Items 7 (*λ* = 0.348) and 14 (*λ* = 0.175). The detailed factor structure, including factor loadings and covariance, is shown in Figure 4.

## 4 Discussion

A total of 22 factor structures from the extant literature were evaluated on HADS‐D [35] in a comprehensive sample of oncology patients close to the time of diagnosis. The aim of the study was to compare the models and identify the optimized model, which would have implications for practice.

The testing of the single‐factorial model [40] yielded the lowest model fit among all models examined. This finding is consistent with the results of Zeilinger et al. [34], who also obtained a poor model fit using a single‐factorial model.

A total of 10 two‐factorial models [31, 34, 35, 36, 42, 43, 44, 45] were examined, and the results obtained were consistent with those reported by Lloyd et al. [66]. Models V [42] and VI [44] also exhibited a high model fit in the present studies, comparable to the original factor structure (Model III; [31, 35, 36]) of the HADS. However, a reliability analysis of the models was not conducted in the corresponding studies [42, 44], precluding a direct comparison with the present results. The findings of the present study indicate that the variance and reliability, as well as the MI grouped by gender and diagnosis, have been demonstrated to achieve reliable values for the original factor structure of the HADS. These results thus support the conclusions previously reported in the literature [34, 66, 67].

The analysis of seven three‐factorial models [47, 48, 49, 50, 51, 52, 53] revealed a satisfactory model fit for each individual model. Nevertheless, due to their relatively lower model quality compared to the final selected model, a more in‐depth examination of these models was not conducted. Notably, the literature suggests that models based on tripartite theory (see ESM Information Box 1; [46]) may not be suitable for application to the HADS [34]. Furthermore, despite an adequate model fit, the reliability of the subscales within these models is compromised by low internal consistency [66].

A detailed examination of the two bifactorial models [56] included in the analysis reveals a satisfactory model fit based on CFI, TLI, and RMSEA. This is evident in the factor loadings and covariances. In both models, the overarching factor achieved loadings ranging from acceptable to very good. Similar to the results of Lloyd et al. [66], Items 7 and 11 in the bifactorial model with the original factor structure (model XVII.i) exhibited comparatively lower loadings than the other items of the factor anxiety. Notably, in contrast to the findings of Norton et al. [56], the present results do not support an explicit relationship between anxiety and depression factors and the third factor restlessness in the bifactorial model. Overall, the results demonstrate that the bifactorial models offer a superior explanation of the factor structure compared to the original factor structure. This improvement is also evident in the total variance of the individual models, which increased over the period between diagnosis and the initial HADS‐D survey.

The results of the differentiation of patients into low vs. moderate or into moderate versus high psychological distress using the total sum score (HADS‐T) revealed a lack of invariance for all three models considered in detail, with the exception of the configural‐metric MI of Model XVII.i. This discrepancy can be attributed, in part, to the possible theoretical assignment to two or three factors. The underlying test theory approach reflects anxiety and depression by evaluating the individual items. This evaluation was carried out by differentiating the total sum score (equivalent to the overarching factor in Models XVII.i and XVII.ii) into low and moderate or moderate and high groups. Differentiating the total sum score causes the initial assessment of anxiety and depression to become less relevant, allowing outliers in the anxiety or depression scale to justify the total load. Notwithstanding the lack of MI, Model XVII.ii showed an acceptable model fit for configural measurement, whereby only MI from configural to metric or metric to scaled was absent. However, no comparable results can be found in the literature because none of the numerous previous studies have grouped using the total sum score.

## 5 Implications

### 5.1 Clinical Implications

From a clinical perspective, these results have important clinical implications for diagnosing and treating cancer patients. Using the bifactorial model with a superordinate factor and original factor structure (Model XVII.i) may improve anxiety and depression diagnosis in cancer patients. However, using the total sum score to categorize patients as experiencing low, moderate, or high psychological distress may introduce potential variance in the measurement. Given the widespread use of the HADS [38, 57, 59, 60, 62] and the total sum score for screening psychological distress [35], the authors of this paper do not argue against using the total sum score. The factor loadings of the overarching factor, in particular, indicate reliable measurement.

Moreover, the present results lend support to the utilization of the total sum score as a more valid measure in terms of the transdiagnostic approach to measuring general distress in cancer patients, thereby corroborating the results of studies [70, 91] on the use of the HADS in other clinical pictures. Consequently, the utilization of the total sum score (as an indicator of the overarching factor of anxiety and depression) reinforces the implementation of the transdiagnostic approach in clinical practice [72] and encourages the use of existing screening instruments such as the HADS (cf. [92]) for the treatment of anxiety and depression as comorbidities of oncological diseases.

### 5.2 Research Implications

This study provides crucial insights into the factor structure of the German version of the Hospital Anxiety and Depression Scale (HADS‐D) in cancer patients, primarily replicating the previously conducted investigation of the HADS factor structure, but this time using the German questionnaire version. Beyond clinical implications, the results of this study confirm the preservation of the original HADS subscales. Although bifactorial models are superior, the use of the original anxiety and depression subscales with their corresponding cut‐offs is still recommended. These findings should be considered in future studies, particularly those using the German HADS version, and align with the results of other authors [56, 66].

## 6 Limitation

The results of this study should be viewed in the context of using secondary data. Although the data were used with a “research question‐driven” approach, for which the current data set was suitable [93], the transparent merging of the two primary data sources into a combined data set shows that much of the data set was excluded from the evaluation due to missing data. However, there are literature‐supported justifications for choosing the “Listwise Deletion” method (exclusion of incomplete cases) in generating the final dataset. It is well known that the Full‐information maximum likelihood (FIML) method is a useful procedure in SEM for imputing missing data, but this requires the fulfillment of the missing at random (MAR) assumption and multivariate normality for the joint distribution of all variables (cf. [94]). Unfortunately, both assumptions, particularly, the MAR assumption, could not be met due to the age of the data and the time frame of data collection, as well as the fact that the data were collected in the context of routine care. Furthermore, the cases in the final dataset are sufficient, considering the minimum case count per item or latent construct, as discussed in the literature (cf. [95]). Therefore, CFA was carried out without imputing missing data. While this conservative approach yielded more reliable results based on real patient data, it also resulted in the loss of potentially relevant cases. Additionally, considering only three models, despite the theory‐based analysis, means that potentially relevant results may be overlooked by omitting analyses of the remaining 19 models. Therefore, the work makes no claim to absolute completeness. In addition, no control group was used, which makes it difficult to interpret the results in comparison with other studies. The study only examines the factor structure of the HADS around the time of diagnosis, but no potential influences of therapy or similar or long‐term effects. To enhance the generalizability of the results, subsequent studies should consider specific oncological diagnosis groups, as well as long‐term survivors and the potential impact of therapy during the screening period.

## 7 Conclusion

To the best of the authors' knowledge, the present study is the first of its kind to comprehensively test the variety of factor structures published and discussed in the literature on the German‐language version of the HADS using a large clinical sample. The findings of the study suggest that the utilization of the original scales for measuring anxiety and depression, inclusive of the associated cut‐offs, is recommended. Moreover, the study validates the implementation of the HADS total sum score as an operationalization of the overarching factor within the context of the recommended bifactorial model.

## Author Contributions

**Thilo Dietz:** conceptualization, formal analysis, methodology, software, visualization, writing – original draft. **Vera Schiewer:** resources, writing – review and editing. **Hildegard Labouvie:** data curation, resources, writing – review and editing. **Anna Hagemeier:** statistical consulting, validation, writing – review and editing. **Irena Pjanic:** writing – review and editing, validation. **Christoph Herrmann‐Lingen:** writing – review and editing, validation. **Michael Kusch:** investigation, methodology, supervision, writing – original draft. All authors approved to the submitted version.

## Funding

The authors have nothing to report.

## Ethics Statement

These data used in this secondary data analysis were collected at the Center for Integrated Oncology (CIO) of the University Hospital Cologne between February 2009 and April 2021. The projects in which the data collection took place were positively evaluated by the Ethics Committee of the Medical Faculty of the University of Cologne under the file numbers AZ 15‐048 and AZ 18‐092.

## Consent

All patients whose data were collected in the context of the primary studies (positive votes from the Ethics Committee of the Medical Faculty of the University of Cologne under the file numbers AZ 15‐048 and AZ 18‐092) provided their informed consent for data collection and processing.

## Conflicts of Interest

C.H.L. is receiving royalties from Hogrefe Publishers for the HADS‐D. He has received lecture honoraria from Novartis and from various hospitals and educational institutions. His research is funded by the German Ministry of Education and Research (BMBF), the German Research Foundation (DFG), and the EU commission. The other authors declare no conflicts of interest.

## Supporting information


**Supporting Information: Box 1.** Tripartite model of anxiety and depression.
**Table S1:** Confirmatory factor analyses of all models based on the HADS‐D data.
**Table S2:** Sample characteristics.
**Table S3:** Summary of HADS‐D item characteristics.
**Table S4:** Model variances according the analyzed models.
**Table S5:** Measurement invariance of the specific models.
**Table S6:** Reliability according the analyzed models.

## Data Availability

In accordance with the prevailing data protection regulations, the data employed in the secondary data analysis is not to be made publicly available. This is also due to the individual ethics approvals of the individual studies from which the data was synthesized. However, the R scripts and R packages utilized for the calculations can be obtained from the authors upon request.
