# Supplementary material for: Factor Structures of the Hospital Anxiety and Depression Scale in Cancer: Implications of Comprehensive Confirmatory Analysis on the German Version of the HADS in an Oncological Sample
Source: Cancer Med. 2026 Jun 1;15(6):e71992. doi: 10.1002/cam4.71992 (PMC13239859; doi:10.1002/cam4.71992)
Supplement: Supplementary file 1 — Supporting Information: Box 1. Tripartite model of anxiety and depression. Table S1: Confirmatory factor analyses of all models based on the HADS‐D data. Table S2: Sample characteristics. Table S3: Summary of HADS‐D item characteristics. Table S4: Model variances according the analyzed models. Table S5: Measurement invariance of the specific models. Table S6: Reliability according the analyzed models. [file CAM4-15-e71992-s001.docx]

# Supplementary Material

**ESM Information box 1**

| **Tripartite model of anxiety and depression**  Clark and Watson (1) proposed the tripartite model of anxiety and depression to explain the high comorbidity between anxious and depressive symptoms and disorders, which ranges from 57% to 70% (2, 3). The model categorizes symptoms into three groups: (a) *Negative Affect* (NA), (b) *Positive Affect* (PA), and (c) *Physiological Hyperarousal* (PH). These dimensions help to distinguish shared and unique features of anxiety and depression.  Negative Affect (NA) is the common factor underlying both anxiety and depression. NA is defined as a "*general dimension of subjective distress and unpleasurable engagement that subsumes a variety of aversive mood states, including anger, contempt, disgust, guilt, fear, and nervousness, with low NA being a state of calmness and serenity*" (4). In the literature, NA is also associated with concepts such as psychological stress or general/emotional distress(5).  Positive Affect (PA) reflects the degree of pleasurable engagement with the environment. High PA includes enthusiasm, energy, mental alertness, interest, joy, social dominance, initiative, and activity (6, 1). A low level of PA, or its absence, is referred to as anhedonia, a core symptom of depression (7, 8).  Physiological Hyperarousal (PH) is defined by increased sympathetic nervous system activity in response to threat, manifesting in somatic symptoms such as shortness of breath, dizziness, lightheadedness, and gastrointestinal disturbances(1). According to the model, PH is considered typical and relatively specific to anxiety disorders (9), although it may also occur in depression (10).  Watson's tripartite model is thus based on (1) *a general distress or negative affect factor common to both anxiety and depression*, (2) *a physiological hyperarousal factor relatively specific to anxiety*, and (3) *an anhedonia/low positive affect factor relatively specific to depression*. This framework provides the theoretical basis for exploratory factor analyses of the HADS (Hospital Anxiety and Depression Scale) (11–13). |
| --- |

References

1. Clark LA, Watson D. Tripartite model of anxiety and depression: psychometric evidence and taxonomic implications. J Abnorm Psychol 1991; 100(3):316–36.

2. Brown C, Schulberg HC, Madonia MJ, Shear MK, Houck PR. Treatment outcomes for primary care patients with major depression and lifetime anxiety disorders. Am J Psychiatry 1996; 153(10):1293–300.

3. Mineka S, Watson D, Clark LA. Comorbidity of anxiety and unipolar mood disorders. Annu Rev Psychol 1998; 49:377–412.

4. Watson D, Clark LA, Tellegen A. Development and validation of brief measures of positive and negative affect: the PANAS scales. J Pers Soc Psychol 1988; 54(6):1063–70.

5. Watson D, Pennebaker JW. Health complaints, stress, and distress: exploring the central role of negative affectivity. Psychol Rev 1989; 96(2):234–54.

6. Anderson ER, Hope DA. A review of the tripartite model for understanding the link between anxiety and depression in youth. Clin Psychol Rev 2008; 28(2):275–87.

7. Joiner TE, Brown JS, Metalsky GI. A test of the tripartite model's prediction of anhedonia's specificity to depression: patients with major depression versus patients with schizophrenia. Psychiatry Res 2003; 119(3):243–50.

8. Watson D, Naragon-Gainey K. On the Specificity of Positive Emotional Dysfunction in Psychopathology: Evidence from the Mood and Anxiety Disorders and Schizophrenia/Schizotypy. Clin Psychol Rev 2009; 30(7):839–48.

9. Joiner TE, Steer RA, Beck AT, Schmidt NB, Rudd MD, Catanzaro SJ. Physiological hyperarousal: construct validity of a central aspect of the tripartite model of depression and anxiety. J Abnorm Psychol 1999; 108(2):290–8.

10. Greaves-Lord K, Ferdinand RF, Sondeijker FEPL, Dietrich A, Oldehinkel AJ, Rosmalen JGM et al. Testing the tripartite model in young adolescents: is hyperarousal specific for anxiety and not depression? J Affect Disord 2007; 102(1-3):55–63.

11. Dunbar M, Ford G, Hunt K, Der G. A confirmatory factor analysis of the Hospital Anxiety and Depression scale: comparing empirically and theoretically derived structures. Br J Clin Psychol 2000; 39(1):79–94.

12. Norton S, Cosco T, Doyle F, Done J, Sacker A. The Hospital Anxiety and Depression Scale: a meta confirmatory factor analysis. J Psychosom Res 2013; 74(1):74–81.

13. Lloyd M, Sugden N, Thomas M, McGrath A, Skilbeck C. The structure of the Hospital Anxiety and Depression Scale: Theoretical and methodological considerations. Br J Psychol 2023; 114(2):457–75.

**ESM Table E1:** Confirmatory factor analyses of all models based on the HADS-D data

|  |  | **SG I (N = 2131)** | | | | | **SG II (N = 2945)** | | | | | **SG III (N = 3236)** | | | | | **SG IV (N = 3395)** | | | | |
| --- | --- | --- | --- | --- | --- | --- | --- | --- | --- | --- | --- | --- | --- | --- | --- | --- | --- | --- | --- | --- | --- |
| **Model** | **No. of factors** | **χ²** | **(df)** | **CFI** | **TLI** | **RMSEA**  **(p)** | **χ²** | **(df)** | **CFI** | **TLI** | **RMSEA**  **(p)** | **χ²** | **(df)** | **CFI** | **TLI** | **RMSEA**  **(p)** | **χ²** | **(df)** | **CFI** | **TLI** | **RMSEA**  **(p)** |
| I | One factorial | 2444.87 | (77) | .937 | .926 | .120*** | 3370.67 | (77) | .939 | .928 | .121*** | 3681.34 | (77) | .94 | .929 | .12*** | 3867.03 | (77) | .94 | .93 | .12*** |
| II | Two factorial | 1153.32 | (76) | .971 | .966 | .082*** | 1712.9 | (76) | .97 | .964 | .086*** | 1875.06 | (76) | .97 | .964 | .086*** | 1965.75 | (76) | .97 | .964 | .086*** |
| III.i |  | 634.13 | (64) | .983 | .979 | .065*** | 893.22 | (64) | .983 | 979 | .066*** | 1015.55 | (64) | .982 | 978 | .068*** | 1049.17 | (64) | .983 | .979 | .067*** |
| III.ii |  | 580.39 | (53) | .984 | .980 | .068*** | 813.68 | (53) | .984 | .979 | .07*** | 921.68 | (53) | .983 | .979 | .071*** | 947.95 | (53) | .984 | .979 | .071*** |
| IV |  | 814.93 | (75) | .98 | .976 | .061*** | 1171.47 | (75) | .98 | .975 | .07*** | 1309.45 | (75) | .979 | .975 | .071*** | 1351.12 | (75) | .98 | .976 | .071*** |
| V |  | 842.94 | (53) | .977 | .972 | .084*** | 1312.3 | (53) | .975 | .968 | .090*** | 1428.86 | (53) | .975 | .969 | .09*** | 1496.23 | (53) | .976 | .97 | .09*** |
| VI |  | 476.28 | (53) | .987 | .983 | .061*** | 694.73 | (53) | .986 | .982 | .064*** | 776.69 | (53) | .986 | .982 | .065*** | 812.12 | (53) | .986 | .982 | .065*** |
| VII.i |  | 2162.71 | (76) | .945 | .934 | .114*** | 3014.78 | (76) | .945 | .935 | .115*** | 3311.93 | (76) | .946 | .935 | .115*** | 3488.38 | (76) | .946 | .936 | .115*** |
| VII.ii |  | 1057 | (76) | .974 | .969 | .078*** | 1428.52 | (76) | .975 | .97 | .078*** | 1605.42 | (76) | .974 | .969 | .079*** | 1654.04 | (76) | .975 | .97 | .078*** |
| VII.iii |  | 1995.78 | (76) | .949 | .939 | .109*** | 2732.89 | (76) | .951 | .941 | .109*** | 2992.68 | (76) | .951 | .942 | .109*** | 3118.94 | (76) | .952 | .943 | .109*** |
| VII.iv |  | 2138.82 | (76) | .945 | .935 | .113*** | 2916.13 | (76) | .974 | .937 | .113*** | 3150.27 | (76) | .949 | .939 | .112*** | 3293.86 | (76) | .949 | .939 | .112*** |
| VIII | Three factorial | 1006.75 | (74) | .975 | .970 | .077*** | 1440.27 | (74) | .975 | .969 | .079*** | 1568.31 | (74) | .975 | .969 | .079*** | 1641.53 | (74) | .975 | .97 | .079*** |
| IX |  | 900.39 | (74) | .978 | .973 | .072*** | 1298.04 | (74) | .977 | .972 | .075*** | 1453.43 | (74) | .977 | .972 | .076*** | 1511.65 | (74) | .977 | .972 | .076*** |
| X |  | 858.61 | (73) | .979 | .974 | .071*** | 1151.07 | (73) | .980 | .975 | .071*** | 1266.52 | (73) | .98 | .975 | .071*** | 1344.3 | (73) | .98 | 975 | .072*** |
| XI |  | 1147.2 | (74) | .972 | .965 | .083*** | 1498.5 | (74) | .974 | .967 | .081*** | 1645.62 | (74) | .974 | .968 | .081*** | 1748.25 | (74) | .974 | .968 | .082*** |
| XII |  | 772.19 | (62) | .978 | .973 | .073*** | 1025.56 | (62) | .979 | .974 | .073*** | 1120.69 | (62) | .980 | .974 | .073*** | 1179.56 | (62) | .98 | .975 | .073*** |
| XIII |  | 887.14 | (74) | .978 | .974 | .072*** | 1193.24 | (74) | .979 | .974 | .072*** | 1336.12 | (74) | .979 | .974 | .073*** | 1403.13 | (74) | .979 | .974 | .073*** |
| XIV |  | 1420.96 | (62) | .963 | .953 | .101*** | 1857.05 | (62) | .966 | .957 | .099*** | 1993.89 | (62) | .967 | .958 | .098*** | 2112.1 | (62) | .967 | .958 | .099*** |
| XV | Four factorial | 1659.37 | (71) | .958 | .946 | .102*** | 2358.59 | (71) | .958 | .946 | .105*** | 2563.45 | (71) | .958 | .947 | .104*** | 2726.29 | (71) | .958 | .947 | .105*** |
| XVI |  | 1014.33 | (58) | .974 | .965 | .088*** | 1512.4 | (58) | .972 | .962 | .092*** | 1640.38 | (58) | .973 | .963 | .092*** | 1729.3 | (58) | .973 | .963 | .092*** |
| XVII.i | Bi-factorial | 535.93 | (62) | .987 | .982 | .06*** | 722.87 | (62) | .988 | .982 | .06*** | 789.99 | (62) | .988 | .982 | .06*** | 825.47 | (62) | .988 | .982 | .06*** |
| XVII.ii |  | 414.11 | (60) | .991 | .986 | **.053**  **(.176)** | 568.46 | (60) | .991 | .986 | **.054**  **(.065)** | 633.12 | (60) | .990 | .985 | .054*  (.03) | 660.5 | (60) | .991 | .986 | .054*  (.028) |

***Note:*** Calculation were performed using the WLSMV estimator with an orthogonal rotation and without data imputation. χ² = Chi-square; df = degrees of freedom; CFI = Comparative Fit Index; TLI = Tucker-Lewis Index; RMSEA = Root Mean Square Error of Approximation (RMSEA); model assignment according to Table 1. Only non-significant P-values (> .001) are reported in the table; *** p < .001; ** p < .01; * p < .05; non-significant values were marked in bold.

**ESM Table E2:** Sample characteristics

|  | **SG I (N = 2131)** | | | | **SG II (N = 2945)** | | | | **SG III (N = 3236)** | | | | **SG IV / Total data set (N = 3395)** | | | |
| --- | --- | --- | --- | --- | --- | --- | --- | --- | --- | --- | --- | --- | --- | --- | --- | --- |
| **Variables** | **N**  **(%)** | **M**  **(SD)** | **MD** | **Range**  **(IQR)** | **N**  **(%)** | **M**  **(SD)** | **MD** | **Range**  **(IQR)** | **N**  **(%)** | **M**  **(SD)** | **MD** | **Range**  **(IQR)** | **N**  **(%)** | **M**  **(SD)** | **MD** | **Range**  **(IQR)** |
| **Age** | 2131  (100) | 55.51  (14.45) | 56 | 18-95  (46-66) | 2945  (100) | 55.65  (14,45) | 57 | 18-95  (47-66) | 3236  (100) | 55.72  (14.36) | 57 | 18-95  (47-66) | 3395  (100%) | 55.73  (14.31) | 57 | 18-96  (47-66) |
| **Gender** |  |  |  |  |  |  |  |  |  |  |  |  |  |  |  |  |
| Male | 841  (39.5) |  |  |  | 1208  (41) |  |  |  | 1354  (41.8) |  |  |  | 1421  (41.9%) |  |  |  |
| Female | 1290  (60.5) |  |  |  | 1737  (59) |  |  |  | 1882  (58.2) |  |  |  | 1974  (58.1%) |  |  |  |
| **Diagnostic groups** |  |  |  |  |  |  |  |  |  |  |  |  |  |  |  |  |
| Malignant neoplasms of digestive organs (*C15–C26*) | 240  (11.3) |  |  |  | 410  (13.9) |  |  |  | 482  (14.9) |  |  |  | 507  (14.9) | - |  | - |
| Malignant neoplasms of respiratory and intrathoracic organs (*C30–C39*) | 128  (6) |  |  |  | 193  (6.6) |  |  |  | 210  (6.5) |  |  |  | 223  (6.6) | - |  | - |
| Melanoma and other malignant neoplasms of skin (*C43–C44*) | 158  (7.4) |  |  |  | 219  (7.4) |  |  |  | 238  (7.4) |  |  |  | 256  (7.5) | - |  | - |
| Malignant neoplasm of breast (*C50*) | 445  (20.9) |  |  |  | 552  (18.8) |  |  |  | 592  (18.3) |  |  |  | 613  (18.1) | - |  | - |
| Malignant neoplasms of female genital organs (*C51–C58*) | 123  (5.8) |  |  |  | 173  (5.9) |  |  |  | 188  (5.8) |  |  |  | 201  (5.9) | - |  | - |
| Malignant neoplasms of eye, brain and other parts of central nervous system (*C69–C72*) | 142  (6.7) |  |  |  | 196  (6.7) |  |  |  | 214  (6.6) |  |  |  | 226  (6.7) | - |  | - |
| Malignant neoplasms of lymphoid, hematopoietic and related tissue (*C81–C96*) | 427  (20.1) |  |  |  | 553  (18.8) |  |  |  | 589  (18.2) |  |  |  | 603  (17.8) | - |  | - |
| Other groups of malignant neoplasms* | 464  (21.8) |  |  |  | 645  (21.9) |  |  |  | 718  (22.2) |  |  |  | 761  (22.4) |  |  |  |
| Other diagnoses (non-malignant neoplasms) | 4  (.2) |  |  |  | 4  (.1) |  |  |  | 5  (.2) |  |  |  | 5  (.1) | - |  | - |
| **Inclusion period of cases** |  |  |  |  |  |  |  |  |  |  |  |  |  |  |  |  |
| Mean difference between initial HADS screening and oncological diagnosis | 2131  (100) | 7.69  (9,00) | 4 | 0-31  (0-13) | 2945  (100) | 20.87  (24.34) | 10 | 0-93  (1-35) | 3236  (100) | 30.7  (39.77) | 13 | 0-182  (2-46) | 3395  (100%) | 41.6  (63.76) | 16 | 0–365  (2-52) |

***Note:*** Diagnosis groups (C00-C14, C40-C41, C45-C49, C60-C63, C64-C68, C73-C75, and C76-C80) summarized due to a frequency < 5% within the individual diagnosis groups. N = number of cases; M = mean; MD = median; SD = standard deviation; Range (minimum to maximum); IQR = inter-quartile range

**ESM Table E3.** Summary of HADS-D item characteristics

|  | **SG I (N = 2131)** | | | **SG II (N = 2945)** | | | **SG III (N = 3236)** | | | **SG IV (N = 3395)** | | |
| --- | --- | --- | --- | --- | --- | --- | --- | --- | --- | --- | --- | --- |
| Item | M (M_SE_) | SK (SK_SE_) | Shapiro-Wilk-Test  (p) | M (M_SE_) | SK (SK_SE_) | Shapiro-Wilk-Test  (p) | M (M_SE_) | SK (SK_SE_) | Shapiro-Wilk-Test  (p) | M (M_SE_) | SK (SK_SE_) | Shapiro-Wilk-Test  (p) |
| 1 | 1.35  (.02) | .36  (.05) | .857  (< .001) | 1.36  (.02) | .36  (.05) | .857  (< .001) | 1.37  (.02) | .34  (.04) | .858  (< .001) | 1.38  (.02) | .33  (.04) | .859  (< .001) |
| 3 | 1.65  (.02) | -.25  (.05) | .872  (< .001) | 1.65  (.02) | -.24  (.05) | .873  (< .001) | 1.64  (.02) | -.232  (.04) | .873  (< .001) | 1.65  (.02) | -.24  (.04) | .873  (< .001) |
| 5 | 1.37  (.02) | .12  (.05) | .879  (< .001) | 1.39  (.02) | .1  (.05) | .879  (< .001) | 1.39  (.02) | .09  (.04) | .879  (< .001) | 1.40  (.02) | .08  (.04) | .880  (< .001) |
| 7 | 1.19  (.02) | .29  (.05) | .868  (< .001) | 1.21  (.02) | .28  (.05) | .870  (< .001) | 1.22  (.02) | .26  (.04) | .870  (< .001) | 1.23  (.02) | .25  (.04) | .871  (< .001) |
| 9 | 1.09  (.02) | .59  (.05) | .834  (< .001) | 1.1  (.02) | .57  (.05) | .838  (< .001) | 1.10  (.02) | .56  (.04) | .839  (< .001) | 1.11  (.02) | .56  (.04) | .840  (< .001) |
| 11 | 1.12  (.02) | .37  (.05) | .861  (< .001) | 1.13  (.02) | .37  (.05) | .861  (< .001) | 1.12  (.02) | .38  (.04) | .861  (< .001) | 1.13  (.02) | .38  (.04) | .862  (< .001 |
| 13 | .870  (.02) | .76  (.05) | .815  (< .001) | .87  (.02) | .77  (.05) | .815  (< .001) | .87  (.02) | .77  (.04) | .814  (< .001) | .88  (.02) | .76  (.04) | .817  (< .001) |
| 2 | 1.02  (.02) | .62  (.05) | .841  (< .001) | 1.05  (.02) | .58  (.05) | .845  (< .001) | 1.06  (.02) | .57  (.04) | .847  (< .001) | 1.07  (.02) | .57  (.04) | .847  (< .001) |
| 4 | .86  (.02) | .59  (.05) | .822  (< .001) | .88  (.02) | .57  (.05) | .825  (< .001) | .88  (.02) | .57  (.04) | .826  (< .001) | .89  (.02) | .56  (.04) | .828  (< .001) |
| 6 | 1.00  (.02) | .64  (.05) | .833  (< .001) | 1.03  (.02) | .62  (.05) | .837  (< .001) | 1.04  (.02) | .60  (.04) | .839  (< .001) | 1.04  (.02) | .6  (.04) | .840  (< .001) |
| 8 | 1.52  (.02) | .12  (.05) | .875  (< .001) | 1.55  (.02) | .07  (.05) | .876  (< .001) | 1.57  (.02) | .05  (.04) | .876  (< .001) | 1.58  (.02) | .04  (.04) | .876  (< .001) |
| 10 | .53  (.02) | 1.44  (.05) | .668  (< .001) | .57  (.02) | 1.36  (.05) | .685  (< .001) | .58  (.02) | 1.34  (.04) | .690  (< .001) | .58  (.02) | 1,33  (.04) | .692  (< .001) |
| 12 | 1.13  (.02) | .5  (.05) | .853  (< .001) | 1.17  (.02) | .44  (.05) | .859  (< .001) | 1.08  (.02) | .42  (.04) | .860  (< .001) | 1.19  (.02) | .40  (.04) | .862  (< .001) |
| 14 | .63  (.02) | 1.25  (.05) | .731  (< .001) | .65  (.02) | 1.21  (.05) | .739  (< .001) | .66  (.02) | 1.20  (.04) | .740  (< .001) | .66  (.02) | 1.2  (.04) | .741  (< .001) |

***Note:*** M = mean; M_SE_ = standard error of the mean; SK = Skewness; SK_SE_ = standard error of the skewness

**ESM Table E4:** Model variance according to the analyzed models

|  | **SG I (N = 2131)** | | | **SG II (N = 2945)** | | | **SG III (N = 3236)** | | | **SG IV (N = 3395)** | | |
| --- | --- | --- | --- | --- | --- | --- | --- | --- | --- | --- | --- | --- |
| **Model / Item-No.** | **III** | **XVII.i** | **XVII.ii** | **III** | **XVII.i** | **XVII.ii** | **III** | **XVII.i** | **XVII.ii** | **III** | **XVII.i** | **XVII.ii** |
| *I1* | .518 | .501 | .501 | .537 | .516 | .513 | .546 | .523 | .521 | .542 | .52 | .518 |
| *I3* | .634 | .764 | .753 | .63 | .754 | .753 | .629 | .762 | .764 | .625 | .762 | .764 |
| *I5* | .708 | .673 | .67 | .7 | .688 | .681 | .698 | .684 | .678 | .701 | .68 | .675 |
| *I7* | .672 | .855 | .882 | .678 | .86 | .864 | .681 | .861 | .859 | .682 | .86 | .858 |
| *I9* | .559 | .722 | .722 | .571 | .717 | .718 | .571 | .715 | .716 | .568 | .716 | .716 |
| *I11* | .11 | .711 | .729 | .105 | .704 | .718 | .108 | .702 | .715 | .112 | .701 | .714 |
| *I13* | .594 | .628 | .685 | .606 | .649 | .703 | .603 | .646 | .706 | .610 | .645 | .699 |
| *I2* | .737 | .494 | .525 | .734 | .485 | .514 | .739 | .494 | .524 | .739 | .49 | .519 |
| *I4* | .771 | .594 | .596 | .769 | .614 | .614 | .766 | .61 | .611 | .768 | .607 | .608 |
| *I6* | .706 | .348 | .364 | .702 | .37 | .387 | .698 | .371 | .385 | .697 | .373 | .388 |
| *I8* | .415 | .159 | .333 | .414 | .134 | .343 | .411 | .136 | .33 | .412 | .146 | .348 |
| *I10* | .329 | .625 | .632 | .344 | .626 | .632 | .341 | .631 | .637 | .343 | .632 | .638 |
| *I12* | .624 | .652 | .656 | .626 | .664 | .669 | .631 | .668 | .672 | .632 | .675 | .679 |
| *I14* | .575 | .589 | .563 | .595 | .614 | .594 | .594 | .606 | .588 | .594 | .605 | .586 |
| *Total variance* | .568 | .594 | .615 | .572 | .600 | .622 | .573 | .601 | .622 | .573 | .601 | .622 |

**Note:** The total variance is calculated by dividing the sum of the individual variances of the items by the number of items ($R^{2}=1-((I1+\ldots+I14)/14$) variance and measurement invariance of the models).

**ESM Table E5:** Measurement invariance of the specific models (total data set)

| **Model** | **Group** |  | **Model level** | | | | | | **Model comparison** | | | | | |
| --- | --- | --- | --- | --- | --- | --- | --- | --- | --- | --- | --- | --- | --- | --- |
|  |  |  | **χ²** | **df** | **p** | **CFI** | **RMSEA** | **p** | **∆χ²** | **df** | **p** | **∆CFI** | **∆RMSEA** | **Decision** |
| **III** | *Gender* | male (N=1974) | - | - | - |  |  |  |  |  |  |  |  |  |
|  |  | female (N=1421) | - | - | - |  |  |  |  |  |  |  |  |  |
|  |  | configural invariance | 2047.889 | 152 | < .001 | .97 | .086 | < .001 |  |  |  |  |  | Acceptable model fit |
|  |  | metric (weak) invariance | 1224.423 | 164 | < .001 | .983 | .062 | < .001 | 13.999 | 12 | .301 | .013 | -.024 | Invariance given |
|  |  | scalar (strong) invariance | 1726.365 | 190 | < .001 | .976 | .069 | < .001 | 67.809 | 26 | < .001 | -.008 | .007 | Invariance given |
|  | *diagnosis* | C50 (N=613) | - | - | - |  |  |  |  |  |  |  |  |  |
|  |  | C81-C96 (N=603) | - | - | - |  |  |  |  |  |  |  |  |  |
|  |  | configural invariance | 773.708 | 152 | < .001 | .969 | .082 | < .001 |  |  |  |  |  | Acceptable model fit |
|  |  | metric (weak) invariance | 717.272 | 164 | < .001 | .973 | .075 | < .001 | 71.452 | 12 | < .001 | .004 | -.007 | Invariance given |
|  |  | scalar (strong) invariance | 881.587 | 190 | < .001 | .966 | .077 | < .001 | -10.395 | 26 | 1.000 | -.007 | .003 | Invariance given |
|  | *HADS* | low distress (N=1460) | - | - | - |  |  |  |  |  |  |  |  |  |
|  |  | moderate distress (N=1064) | - | - | - |  |  |  |  |  |  |  |  |  |
|  |  | configural invariance | 1291.103 | 152 | < .001 | .812 | .077 | < .001 |  |  |  |  |  | Inacceptable model fit |
|  |  | metric (weak) invariance | 1410.833 | 164 | < .001 | .794 | .078 | < .001 | 145.97 | 12 | < .001 | -.018 | .001 | Invariance not given |
|  |  | scalar (strong) invariance | 1629.29 | 190 | < .001 | .763 | .078 | < .001 | 237.9 | 26 | < .001 | -.032 | 0 | Invariance not given |
|  |  | moderate distress (N=1064) | - | - | - |  |  |  |  |  |  |  |  |  |
|  |  | high distress (N=871) | - | - | - |  |  |  |  |  |  |  |  |  |
|  |  | configural invariance | 1295.569 | 152 | < .001 | .723 | .088 | < .001 |  |  |  |  |  | Inacceptable model fit |
|  |  | metric (weak) invariance | 1360.196 | 164 | < .001 | .711 | .087 | < .001 | 114.41 | 12 | < .001 | -.012 | -.001 | Invariance not given |
|  |  | scalar (strong) invariance | 1638.867 | 190 | < .001 | .65 | .089 | < .001 | 299.13 | 26 | < .001 | -.061 | .002 | Reject |
| **XVII.i** | *Gender* | male (N=1974) | - | - | - |  |  |  |  |  |  |  |  |  |
|  |  | female (N=1421) | - | - | - |  |  |  |  |  |  |  |  |  |
|  |  | configural invariance | 912.35 | 124 | < .001 | .988 | .061 | < .001 |  |  |  |  |  | Acceptable model fit |
|  |  | metric (weak) invariance | 523.407 | 149 | < .001 | .994 | ,038 | 1,00 | 29,585 | 25 | ,240 | ,006 | -,023 | Invariance given |
|  |  | scalar (strong) invariance | 768.929 | 174 | < .001 | .991 | ,045 | ,995 | 81,453 | 25 | < .001 | -,003 | ,006 | Invariance given |
|  | *diagnosis* | C50 (N=613) | - | - | - |  |  |  |  |  |  |  |  |  |
|  |  | C81-C96 (N=603) | - | - | - |  |  |  |  |  |  |  |  |  |
|  |  | configural invariance | 326.611 | 124 | .021 | .990 | .052 | .316 |  |  |  |  |  | Acceptable model fit |
|  |  | metric (weak) invariance | 429.533 | 149 | < .001 | .986 | .056 | .062 | 108.95 | 25 | < .001 | -.004 | .004 | Invariance given |
|  |  | scalar (strong) invariance | 522.152 | 174 | < .001 | .983 | .057 | .016 | 12.028 | 25 | .986 | -.003 | .002 | Invariance given |
|  | *HADS* | low distress (N=1460) | - | - | - |  |  |  |  |  |  |  |  |  |
|  |  | moderate distress (N=1064) | - | - | - |  |  |  |  |  |  |  |  |  |
|  |  | configural invariance | 637.712 | 124 | < .001 | .915 | .057 | .003 |  |  |  |  |  | Acceptable model fit |
|  |  | metric (weak) invariance | 967.681 | 149 | < .001 | .865 | .066 | < .001 | 307,64 | 25 | < .001 | -.005 | .009 | Invariance given |
|  |  | scalar (strong) invariance | 1141.719 | 174 | < .001 | .840 | .066 | < .001 | 197,01 | 25 | < .001 | -.025 | 0 | Invariance not given |
|  |  | moderate distress (N=1064) | - | - | - |  |  |  |  |  |  |  |  |  |
|  |  | high distress (N=871) | - | - | - |  |  |  |  |  |  |  |  |  |
|  |  | configural invariance | 565.394 | 124 | < .001 | .893 | .061 | < .001 |  |  |  |  |  | Inacceptable model fit |
|  |  | metric (weak) invariance | 860.441 | 149 | < .001 | .828 | .070 | < .001 | 290 | 25 | < .001 | -.065 | .009 | Invariance not given |
|  |  | scalar (strong) invariance | 1089.133 | 174 | < .001 | .779 | .074 | < .001 | 222.94 | 25 | < .001 | -.049 | .003 | Invariance not given |
| **XVII.ii** | *Gender* | male (N=1974) | - | - | - |  |  |  |  |  |  |  |  |  |
|  |  | female (N=1421) | - | - | - |  |  |  |  |  |  |  |  |  |
|  |  | configural invariance | 733.779 | 120 | < .001 | .990 | .055 | .017 |  |  |  |  |  | Acceptable model fit |
|  |  | metric (weak) invariance | 461.077 | 144 | < .001 | .995 | .036 | 1.00 | 33.712 | 24 | .09 | .005 | -.019 | Invariance given |
|  |  | scalar (strong) invariance | 668.456 | 168 | < .001 | .992 | .042 | 1.00 | 75.816 | 24 | < .001 | -.003 | .006 | Invariance given |
|  | *diagnosis* | C50 (N=613) | - | - | - |  |  |  |  |  |  |  |  |  |
|  |  | C81-C96 (N=603) | - | - | - |  |  |  |  |  |  |  |  |  |
|  |  | configural invariance | 283.854 | 120 | .290 | .992 | .047 | .715 |  |  |  |  |  | Acceptable model fit |
|  |  | metric (weak) invariance | 425.315 | 144 | < .001 | .986 | .057 | .037 | 133.56 | 24 | < .001 | -.006 | .01 | Invariance given |
|  |  | scalar (strong) invariance | 457.175 | 168 | < .001 | .986 | .053 | .175 | 26.201 | 24 | .343 | 0 | -.003 | Invariance given |
|  | *HADS* | low distress (N=1460) | - | - | - |  |  |  |  |  |  |  |  |  |
|  |  | moderate distress (N=1064) | - | - | - |  |  |  |  |  |  |  |  |  |
|  |  | configural invariance | 591.856 | 120 | < .001 | .922 | .056 | .016 |  |  |  |  |  | Acceptable model fit |
|  |  | metric (weak) invariance | NA | NA | NA | NA | NA | NA | NA | NA | NA | NA | NA | Invariance not given |
|  |  | scalar (strong) invariance | NA | NA | NA | NA | NA | NA | NA | NA | NA | NA | NA | Invariance not given |
|  |  | moderate distress (N=1064) | - | - | - |  |  |  |  |  |  |  |  |  |
|  |  | high distress (N=871) | - | - | - |  |  |  |  |  |  |  |  |  |
|  |  | configural invariance | 503.784 | 120 | < .001 | .907 | .058 | .008 |  |  |  |  |  | Acceptable model fit |
|  |  | metric (weak) invariance | 726.264 | 144 | < .001 | .859 | .065 | < .001 | 213.16 | 24 | < .001 | -.048 | .007 | Invariance not given |
|  |  | scalar (strong) invariance | NA | NA | NA | NA | NA | NA | NA | NA | NA | NA | NA | Invariance not given |

**Note:** N = 3395 (Subgroup IV); *the bi-factorial model by Norton et al. (2013) was used as a single-factorial model within the analysis. Δχ² is the difference in chi-square values between the nested model and the configural, metric, and scalar models.

**ESM Table E6:** Reliability according the analyzed models

| **Model**  **(factors)** | **Subscale**  **(Items)** | **SG I**  **(N = 2131)** | **SG II**  **(N = 2945)** | **SG III**  **(N = 3326)** | **SG IV**  **(N = 3395)** |
| --- | --- | --- | --- | --- | --- |
|  |  | **α** | **α** | **α** | **α** |
| II | Anxiety  (I1, I3, I5, I7, I9, I11, I13) | .847 | .848 | .849 | .850 |
|  | Depression  (I2, I4, I6, I8, I10, I12, I14) | .866 | .870 | .869 | .870 |
| XVII.i | Anxiety  (I1, I3, I5, I7, I9, I11, I13) | .847 | .848 | .849 | .850 |
|  | Depression  (I2, I4, I6, I8, I10, I12, I14) | .866 | .870 | .869 | .870 |
|  | Overarching factor  (I1, I2, I3, I4, I5, I6, I7, I8, I9, I10, I11, I12, I13, I14) | .908 | .911 | .911 | .911 |
| XVII.ii | Anxiety  (I1, I3, I5, I9, I13) | .85 | .854 | .854 | .854 |
|  | Depression  (I2, I4, I6, I8, I10, I12) | .866 | .870 | .869 | .870 |
|  | Restlessness  (I7, I11, I14) | .589 | .599 | .603 | .605 |
|  | Overarching factor  (I1, I2, I3, I4, I5, I6, I7, I8, I9, I10, I11, I12, I13, I14) | .908 | .911 | .911 | .911 |
